# Comprehensive Analysis of the Prognostic Role and Mutational Characteristics of m6A-Related Genes in Lung Squamous Cell Carcinoma

**DOI:** 10.3389/fcell.2021.661792

**Published:** 2021-03-25

**Authors:** Chang Gu, Xin Shi, Wenli Qiu, Zhenyu Huang, Yan Yu, Feng Shen, Yumei Chen, Xufeng Pan

**Affiliations:** ^1^Department of Thoracic Surgery, Shanghai Chest Hospital, Shanghai Jiao Tong University, Shanghai, China; ^2^Department of Thoracic Surgery, Shanghai Pulmonary Hospital, Tongji University School of Medicine, Shanghai, China; ^3^Department of Cardiology, Shanghai Chest Hospital, Shanghai Jiao Tong University, Shanghai, China; ^4^Department of Lab Medicine, The Affiliated Hospital of Youjiang Medical University for Nationalities, Baise, China; ^5^Department of Colorectal and Anal Surgery, Xinhua Hospital, Shanghai Jiao Tong University School of Medicine, Shanghai, China; ^6^Shanghai Colorectal Cancer Research Center, Shanghai, China; ^7^School of Biomedical Engineering, Shanghai Jiao Tong University, Shanghai, China; ^8^Department of Nuclear Medicine, Ren Ji Hospital, Shanghai Jiao Tong University School of Medicine, Shanghai, China

**Keywords:** m6A, prognosis, lung squamous cell carcinoma, RNA methylation, nomogram

## Abstract

**Background:**

There have been limited treatment therapies for lung squamous cell carcinoma (LUSC). M6A-related genes may be the next therapeutic targets for LUSC. In this study, we explored the prognostic role and mutational characteristics of m6A-related genes in LUSC.

**Methods:**

LUSC gene expression data, mutational data, and corresponding clinical information were extracted from The Cancer Genome Atlas database. Differentially expressed genes (DEGs) were identified, and the mutation characteristics of LUSC patients were explored. Then, m6A-related genes were extracted and the correlations among the genes were detected. Finally, the prognostic roles of the genes were investigated and the nomogram model was developed. Besides, the protein–protein interaction (PPI) network was used to explore the potential interactions among the genes.

**Results:**

In total, there are 551 LUSC samples enrolled in our study, containing 502 LUSC tumor samples and 49 adjacent normal LUSC samples, respectively. There were 2970 upregulated DEGs and 1806 downregulated DEGs were further explored. IGF2BP1 and RBM15 had significant co-occurrence frequency (*p* < 0.05). Besides, METTL14 and ZC3H13 or YTHDF3 also had significant co-occurrence frequency (*p* < 0.05). All the m6A-related genes represent the positive correlation. WTAP was identified as a prognostic gene in the TCGA database while YTHDC1 and YTHDF1 were identified as prognostic genes. In multivariate Cox analysis, YTHDF1, age, pN stage, pTNM stage, and smoking were all identified as significant prognostic factors for OS.

**Conclusion:**

We investigated the expression patterns and mutational characteristics of LUSC patients and identified three potential independent prognostic m6A-related genes (WTAP, YTHDC1, and YTHDF1) for OS in LUSC patients.

## Introduction

Lung cancer is one of the main causes of cancer-related deaths worldwide, with an approximate 5 year overall survival rate of 16–20% (Gu et al., 2017, 2018a,b, 2020a). Non-small cell lung cancer (NSCLC) accounts for four-fifths of all lung cancer types (Gu et al., 2016, 2018a; Chen et al., 2020). Of these, lung squamous cell carcinoma (LUSC) and lung adenocarcinoma (LUAD) account for the vast majority, reaching about 55 and 30% of all NSCLCs, respectively (Gu et al., 2016). In recent years, there have been many targeted therapies for LUAD, whereas the targeted schemes for LUSC are still limited (Yuan et al., 2017).

N6-Methyladenosine (m6A) RNA modification is defined as an adenosine methylation at the N6 position, identifying as the most abundant mRNA modification, widely discovered in a vast number of eukaryotic species, including yeast, mammals, insects, plants, and certain viruses (Beemon and Keith, 1977; Levis and Penman, 1978; Aloni et al., 1979; Nichols, 1979; Schafer, 1982; Clancy et al., 2002). Besides, m6A modifications exist on almost all types of coding and non-coding RNAs and dynamically regulate their relevant molecular processes and physiological and pathological functions (Gu et al., 2020b). According to the characteristics of m6A proteins, they are divided as “writer,” “eraser,” and “reader” proteins, which dynamically regulate tumor-related pathological and physiological functions (Gu et al., 2020b). Many previous researches have proved the effects of N6-methyladenosine (m6A) RNA modification and its ability to regulate and coordinate related gene expression, whose level will profoundly affect cancer characteristics (Gu et al., 2020b; Shen et al., 2020; Wei et al., 2020). For example, in lung cancer, METTL3 acts as an oncogene, which increases the growth, survival, and invasion of lung adenocarcinoma cells (Lin et al., 2016). This phenomenon suggests that m6A modifications and related genes may play important roles in tumor inhibition or tumorigenesis, which means that m6A modifications or m6A-related genes may be the next tumor therapeutic targets, especially for LUSC (Pei et al., 2020).

Recently, studies on the prognostic role of m6A genes have emerged, including LUAD (Zhang Y. et al., 2020), renal cell carcinoma (Gao et al., 2020), cervical cancer (Yang et al., 2020), colorectal cancer (Sun et al., 2020), and breast cancer (Wei et al., 2020). However, few research focus on LUSC. Herein, we explored the m6A-related genes and the m6A gene-related mutations using The Cancer Genome Atlas (TCGA) databases, attempting to unearth the underlying molecular mechanisms of LUSC tumorigenesis and progression, and then helping develop new and effective targeted therapy regimens.

## Materials and Methods

### Data Source

LUSC gene expression data, mutational data, and corresponding clinical information were obtained from the TCGA database^1^. All the data were available online and defined as open-access. In total, there are 551 LUSC samples were enrolled in our study, containing 502 LUSC tumor samples and 49 adjacent normal LUSC samples, respectively. We conducted the study with the TCGA publication guidelines.

### Differentially Expressed Gene (DEG) Identification

Firstly, 49 LUSC tumor samples and the corresponding 49 adjacent normal LUSC samples were selected. Second, the Ensembl database^2^ was used for identifying the gene IDs. Then, principal component analysis (PCA) was performed to confirm the heterogeneity of the two groups. The PCA graph was obtained. Finally, differentially expressed mRNAs were obtained utilizing the package of “edge R” with absolute log fold change (FC) > 2 and the false discovery rate (FDR)-adjusted *P* < 0.05 by R software (Version 4.0.3) (Gu et al., 2020d). After obtaining the lists of DEGs, the volcano map and corresponding heat map were drawn.

### m6A Gene Extraction

The m6A-related genes are derived from Gu’s review (Gu et al., 2020b) on RNA m6A modification in cancers, including “writer” proteins (METTL3, METTL5, METTL14, METTL16, ZC3H13, RBM15, WTAP, KIAA1429), “reader” proteins (YTHDF1, YTHDF2, YTHDF3, YTHDC1, YTHDC2, HNRNPC, IGF2BP1, IGF2BP2, IGF2BP3), and “eraser” proteins (FTO, ALKBH5).

### Mutational Analyses

The LUSC mutational data were also downloaded from the TCGA database, with samples’ clinical information. In order to identify the somatic mutations of LUSC patients, we analyzed the mutational data and visualized the data using the R package “maftools.” Besides, we further explored the somatic interactions among the m6A genes, along with the status of single-nucleotide polymorphism (SNP) and hypermutated genomic regions (Shi et al., 2018a,b; Zhang L. et al., 2020).

### Survival Analysis and Subtyping

All the mRNA expressions of m6A-related genes were calculated, and patients were separated by the median expression level of each gene (highly expressed group and lowly expressed group). The Kaplan–Meier (KM) survival analyses were used to compare the survival difference between lowly and highly expressed groups based on each m6A-related gene group, with log-rank test. Besides, all the m6A-related genes were validated using Kaplan–Meier Plotter^3^. Then, the univariate and multivariate Cox analyses were performed to predict the prognostic significance for overall survival (OS). R package “ConsensusClusterPlus” was used for consistency analysis, and the maximum number of clusters is 6. Besides, four-fifths of the total sample is drawn 100 times, clusterAlg = “hc,” innerLinkage=“ward D2.” Clustering heat maps were then drawn by R package “pheatmap.” The gene expression heat map retains genes with SD > 0.1. If the number of input genes is more than 1,000, it will extract the top 25% genes after sorting the SD.

### Nomogram for Predicting OS

After obtaining prognostic m6A-related genes in multivariate Cox analysis, all the prognostic genes and relevant clinical characteristics were included for the nomogram modeling, with 1, 2, 3, and 5 survival prediction scores. Then, the calibration curves for each year were also drawn.

### Protein–Protein Interaction (PPI) Network

After identifying the key prognostic m6A-related genes, STRING database^4^ was used for exploring the interactions among the genes (Gu et al., 2020c).

## Results

**FIGURE 1:**
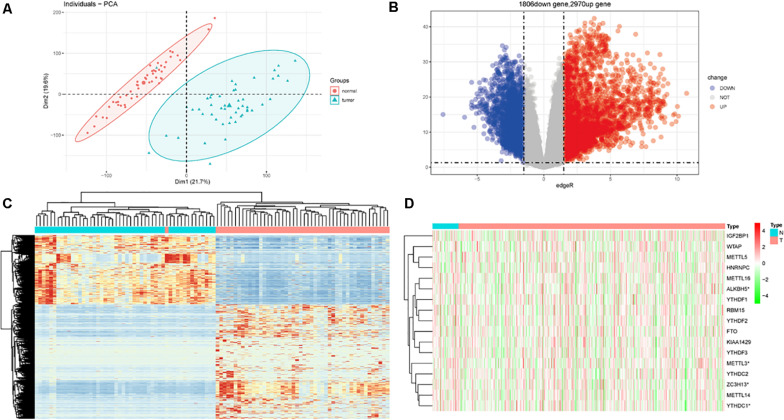
Differentially expressed genes (DEGs) in patients with lung squamous cell carcinoma (LUSC). **(A)** Principal component analysis (PCA) for tumor and normal tissues. **(B)** DEGs between tumor and normal tissues. **(C)** Heat map for DEGs in paired samples. **(D)** The expression of m6A-related genes in total LUSC patients in TCGA database.

In total, there are 551 LUSC samples enrolled in our study, containing 502 LUSC tumor samples and 49 adjacent normal LUSC samples, respectively. The baseline characteristics of the patients were listed (Supplementary Table 1). After matching the paired normal samples for tumor samples, 49 LUSC pairs were finally obtained. The PCA graph showed the significant difference between LUSC tumor and adjacent normal LUSC samples (Figure 1A). There were 2970 upregulated DEGs and 1806 downregulated DEGs further explored (Figure 1B). The heat map of DEGs in the 49 paired samples were also drawn (Figure 1C). Besides, the m6A-related genes were extracted from all the 551 LUSC samples; the expressed patterns are shown in Figure 1D.

**FIGURE 2:**
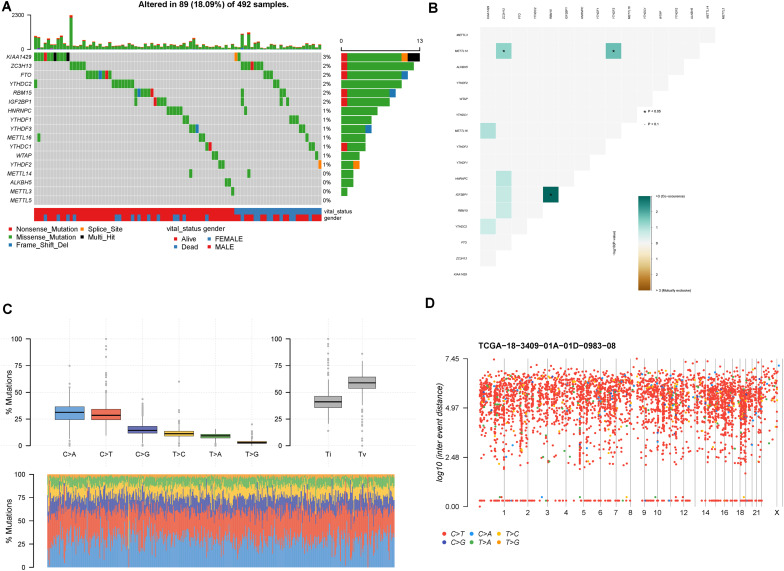
Mutation patterns of LUSC patients. **(A)** Oncoplot displays the mutational patterns of m6A-related genes in 89 LUSC patients. **(B)** The co-expression patterns of m6A-related genes in LUSC patients. **(C)** The SNP patterns of LUSC patients. **(D)** Rainfall plot shows hypermutated genomic regions. *p < 0.05.

In mutational data, 89 patients contained the mutational m6A-related genes in 492 LUSC patients. The top 10 mutational m6A-related genes were KIAA1429 (3%), ZC3H13 (2%), FTO (2%), YTHDC2 (2%), RBM15 (2%), IGF2BP1 (2%), HNRNPC (1%), YTHDF1 (1%), YTHDF3 (1%), and METTL16 (1%), respectively (Figure 2A). Furthermore, the somatic interactions among the m6A genes were detected (Figure 2B). We found that IGF2BP1 and RBM15 had significant co-occurrence frequency (*p* < 0.05). Besides, METTL14 and ZC3H13 or YTHDF3 also had significant co-occurrence frequency (*p* < 0.05). The status of single-nucleotide polymorphism (SNP) and hypermutated genomic regions was also investigated; C>A and C>T were the major two types of mutations, and the convention rates in each samples were also shown in the stacking bar chart (Figure 2C). A rainfall plot showed hypermutated genomic regions according to different SNP mutational types (Figure 2D).

**FIGURE 3:**
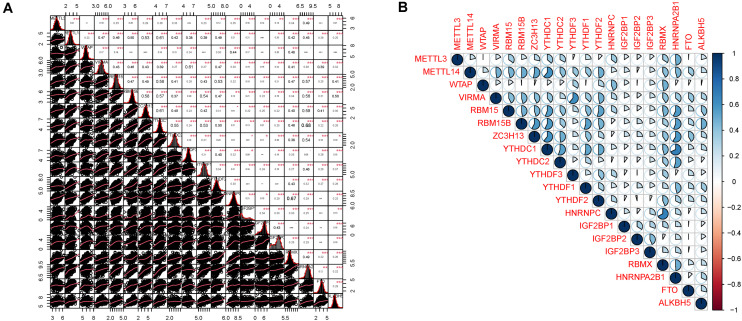
Correlation analysis among m6A related genes. **(A)** The distributions of each sample and the correlation coefficients were calculated. **(B)** The correlation coefficients were drawn by pie charts.

**FIGURE 4:**
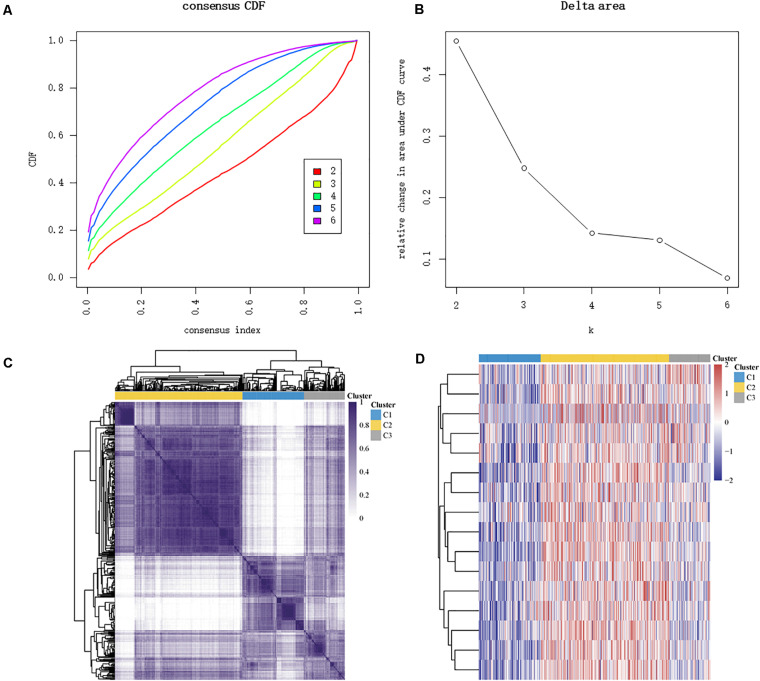
Identification of consensus clusters according to the expression similarity of m6A-related genes. **(A)** Cumulative distribution function (CDF) (*k* = 2–6). **(B)** Relative change in area under the CDF curve (*k* = 2–6). **(C)** The matrix of consensus clustering (*k* = 3). **(D)** Heat map of m6A-related gene expression in different subgroups; red represents high expression while blue represents low expression.

**FIGURE 5:**
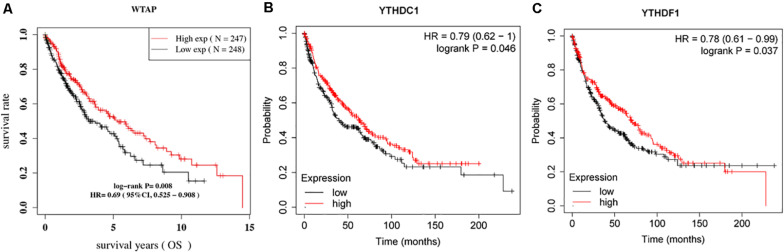
Survival curves according to the expression of **(A)** WTAP; **(B)** YTHDC1; and **(C)** YTHDF1.

The major m6A-related genes were utilized to explore the relationships among these genes by a correlation analysis. All the m6A-related genes represent the positive correlation (Figure 3). In subtyping analyses, we selected *k* = 3 as the cutoff value to develop subtyping groups (Figure 4). Then, we explored the prognostic roles of each m6A-related gene by KM curves with the log-rank test. WTAP (log-rank *p* = 0.012, HR = 0.703, 95% CI = 0.535–0.925) was identified as a prognostic gene in the TCGA database while YTHDC1 (log-rank *p* = 0.046, HR = 0.79, 95% CI = 0.62–1) and YTHDF1 (log-rank *p* = 0.037, HR = 0.78, 95% CI = 0.61–0.99) were identified as prognostic genes in microarray samples using KM Plotter (Figure 5).

**FIGURE 6:**
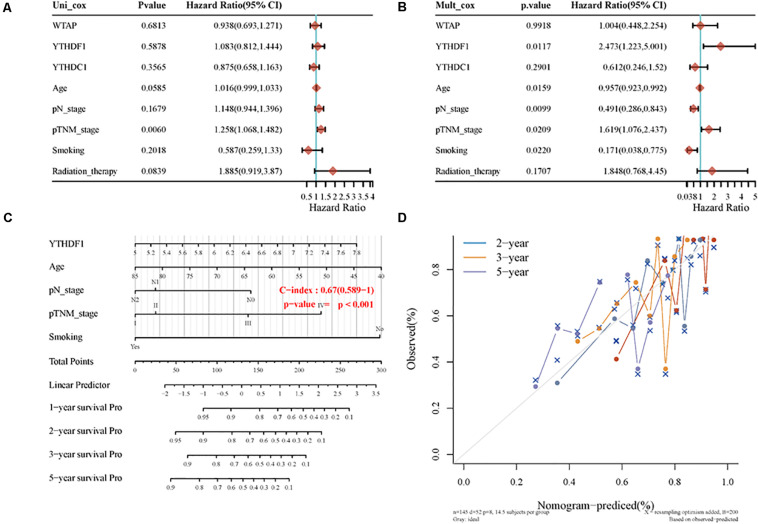
The identification of prognostic factor for OS and the development of the nomogram. **(A)** Univariate Cox analysis. **(B)** Multivariate Cox analysis. **(C)** Nomogram for OS in LUSC patients. **(D)** The calibration curves for each year.

In univariate Cox proportional hazard regression, only the pTNM stage (*p* = 0.0060, HR = 1.258, 95% CI = 1.068–1.482) was identified as a significant prognostic factor for OS while in multivariate Cox proportional hazards regression, YTHDF1 (*p* = 0.0117, HR = 2.473, 95% CI = 1.223–5.001), age (*p* = 0.0159, HR = 0.957, 95% CI = 0.923–0.992), pN stage (*p* = 0.0099, HR = 0.491, 95% CI = 0.286–0.843), pTNM stage (*p* = 0.0209, HR = 1.619, 95% CI = 1.076–2.437), and smoking (*p* = 0.0220, HR = 0.171, 95% CI = 0.038–0.775) were all identified as significant prognostic factors for OS. Afterward, we built a nomogram for LUSC patients using YTHDF1, age, pN stage, and smoking, with the c index as 0.67 (*p* < 0.001, 95% CI 0.589–1) (Figure 6).

After obtaining the three prognostic m6A-related genes, the representative immunohistochemical images (Supplementary Figure 1) and PPI network (Supplementary Figure 2) were explored. Besides, for YTHDF1, the box plot (Supplementary Figure 3) and stage plot (Supplementary Figure 4) were further investigated.

## Discussion

There is limited treatment strategy for LUSC, and the prognosis of LUSC patients remains gloomy. In recent years, targeted therapies and immunotherapy are emerging and shed some light on tumor treatment. Many biomarkers, including specific genes, mRNA/lncRNA/miRNA signatures, and DNA methylation markers, have been identified and used in clinic (Gu and Chen, 2020; Jiao and Yang, 2020). RNA m6A modifications and m6A-related genes were verified as credible markers for different diseases (Gu et al., 2020b). In this article, we explored the prognostic role of m6A-related genes and the mutational status of LUSC patients using the TCGA database, which suggest that WTAP, YTHDC1, and YTHDF1 act as promising prognostic factors and biomarker for LUSC.

In survival analyses, we found that WTAP (log-rank *p* = 0.012, HR = 0.703, 95% CI = 0.535–0.925) were identified as a prognostic gene in the TCGA database while YTHDC1 (log-rank *p* = 0.046, HR = 0.79, 95% CI = 0.62–1) and YTHDF1 (log-rank *p* = 0.037, HR = 0.78, 95% CI = 0.61–0.99) were identified as prognostic genes in microarray samples using KM Plotter. For WTAP, WT1-associated protein, the accumulation of METTL3 and METTL14 requires WTAP, which can bind to the METTL3/METTL14 complex. Besides, WTAP helps recruit optimal substrate and localize the METTL3/METTL14 complex (Zhong et al., 2008; Ping et al., 2014). Yu et al. (2019) demonstrated that, in high-grade serous ovarian carcinoma, WTAP is highly expressed and the higher expression of WTAP is associated with poor survival. After validation in SKOV3 and 3AO cell lines, they found that WTAP may act as a prognostic factor for patients with high-grade serous ovarian carcinoma. YTHDC1, YTH domain-containing proteins 1, regulates the process of mRNA splicing by recruiting and combining serine/arginine-rich splicing factor 3 (SRSF3) in the cell nucleus (Xiao et al., 2016). Moreover, YTHDC1 assists in mRNA exportation from the nucleus to the cytoplasm (Roundtree et al., 2017). In bladder urothelial carcinoma (BLCA), a study screened and validated 9 RNA-binding proteins for prognostic model establishment using the TCGA database and found that YTHDC1 is important for oncogenesis, development, and metastasis in BLCA (Guo et al., 2020). YTHDF1, the YT521-B homology (YTH) domain family protein 1, is reported to recruit translation initiation factors for facilitating translation (Wang et al., 2015). YTHDF1 also has a controversial role in NSCLC; Shi et al. (2019) showed that lack of YTHDF1 inhibits lung adenocarcinoma cell proliferation and tumor formation in xenograft by affecting the translational efficiency of cyclin D1, CDK4, and CDK2 and a high expression level of YTHDF1 is relevant to better prognosis, which is similar to our results.

There are many differences between LUSC and LUAD in m6A-related gene expression patterns. Therefore, there also exist some differences in the significant m6A-related genes for patient outcomes. In LUAD, Zhang Y. et al. (2020) demonstrated that METTL3, YTHDF1, and YTHDF2 were identified as prognostic genes and associated with better relapse-free survival (RFS) and OS. Li et al. (2020) analyzed the TCGA database combined with the Genotype-Tissue Expression (GTEx) database and calculated the prognostic signature-based risk scores of RBM15, HNRNPC, and KIAA1429. They found that the three m6A-related genes not only have strong associations with clinicopathological features and clinical prognosis of LUAD but also act as significant prognostic factors for LUAD. Similarly, Li and Zhan (2020) investigated the m6A-related gene signature for predictive, preventive, and personalized medicine using 511 LUAD samples, 502 LUSC samples, and 109 normal samples obtained from the TCGA database; they found in the whole NSCLC cohort that the three-m6A-related signature (METTL3, KIAA1429, and IGF2BP1) was developed as a prognostic model, which helped in classifying NSCLC patients into low-risk and high-risk groups. In our study, we found that WTAP, YTHDC1, and YTHDF1 were identified as prognostic genes in LUSC, which means that LUAD and LUSC share different prognostic m6A-related gene signatures.

There are some limitations in this study. First, this study is an analysis that uses public databases and lacks some validation by our own cohort; we will further investigate the three m6A-related genes in our own LUSC cohort. Second, the targeted downstream genes of the three genes were not further explored, which may cause some bias in estimating the targeted drugs, which also need further investigation. Third, the number of LUSC patients is still small for selecting the appropriate model and the results still need further validation.

## Conclusion

In conclusion, this study explored the expression patterns and mutational characteristics of LUSC patients and identified three potential independent prognostic m6A-related genes (WTAP, YTHDC1, and YTHDF1) for OS in LUSC patients, which may be helpful for molecular subtyping of LUSC and providing new insight into the potential molecular mechanisms of the ontogenesis, development, and metastasis of LUSC.

## Data Availability Statement

The original contributions presented in the study are included in the article/Supplementary Material, further inquiries can be directed to the corresponding author/s.

## Author Contributions

XP, YC, and FS: conception, design, and administrative support. CG, XS, and WQ: collection and assembly of the data, and data analysis and interpretation. All authors wrote the manuscript and approved the submitted version.

## Conflict of Interest

The authors declare that the research was conducted in the absence of any commercial or financial relationships that could be construed as a potential conflict of interest.

## Footnotes

1 https://portal.gdc.cancer.gov/

2 www.ensembl.org/

3 https://kmplot.com/analysis/

4 https://string-db.org/

## Supplementary Material

Supplementary Figure 1 Representative immunohistochemical images for WTAP and YTHDC1.

Supplementary Figure 2 PPI network for WTAP, YTHDC1, and YTHDF1.

Supplementary Figure 3 The box plot for YTHDF1 in LUSC patients.

Supplementary Figure 4 The stage plot for YTHDF1 in LUSC patients.

Supplementary Table 1 Baseline characteristics of the LUSC patients in TCGA database.
