# Supplementary figures and images for: Comprehensive Analysis of the Prognostic Role and Mutational Characteristics of m6A-Related Genes in Lung Squamous Cell Carcinoma
Source: Front Cell Dev Biol. 2021 Mar 25;9:661792. doi: 10.3389/fcell.2021.661792 (PMC8027321; doi:10.3389/fcell.2021.661792)

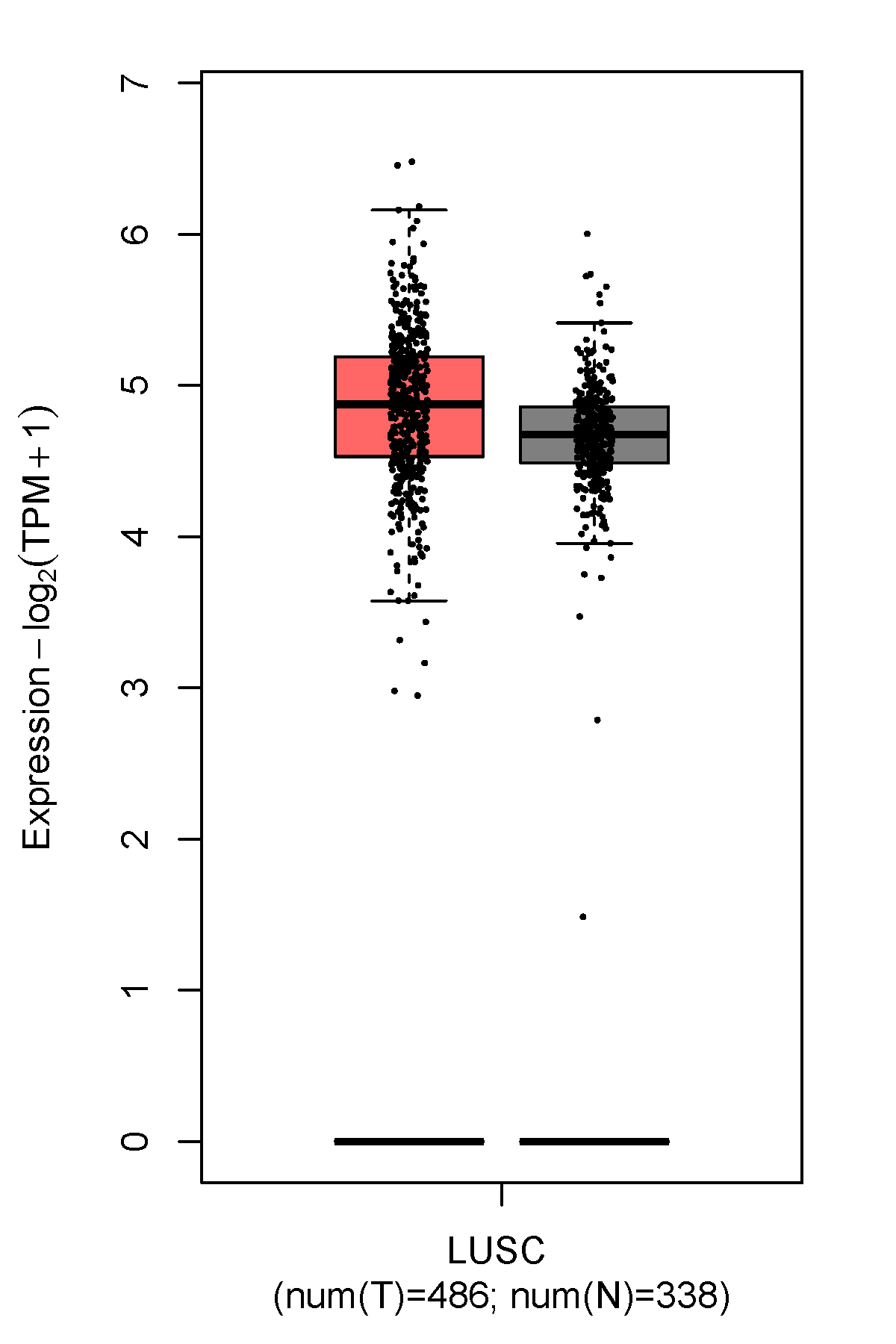

Supplement: Supplementary Figure 1 — Representative immunohistochemical images for WTAP and YTHDC1. [file Image_1.TIFF]

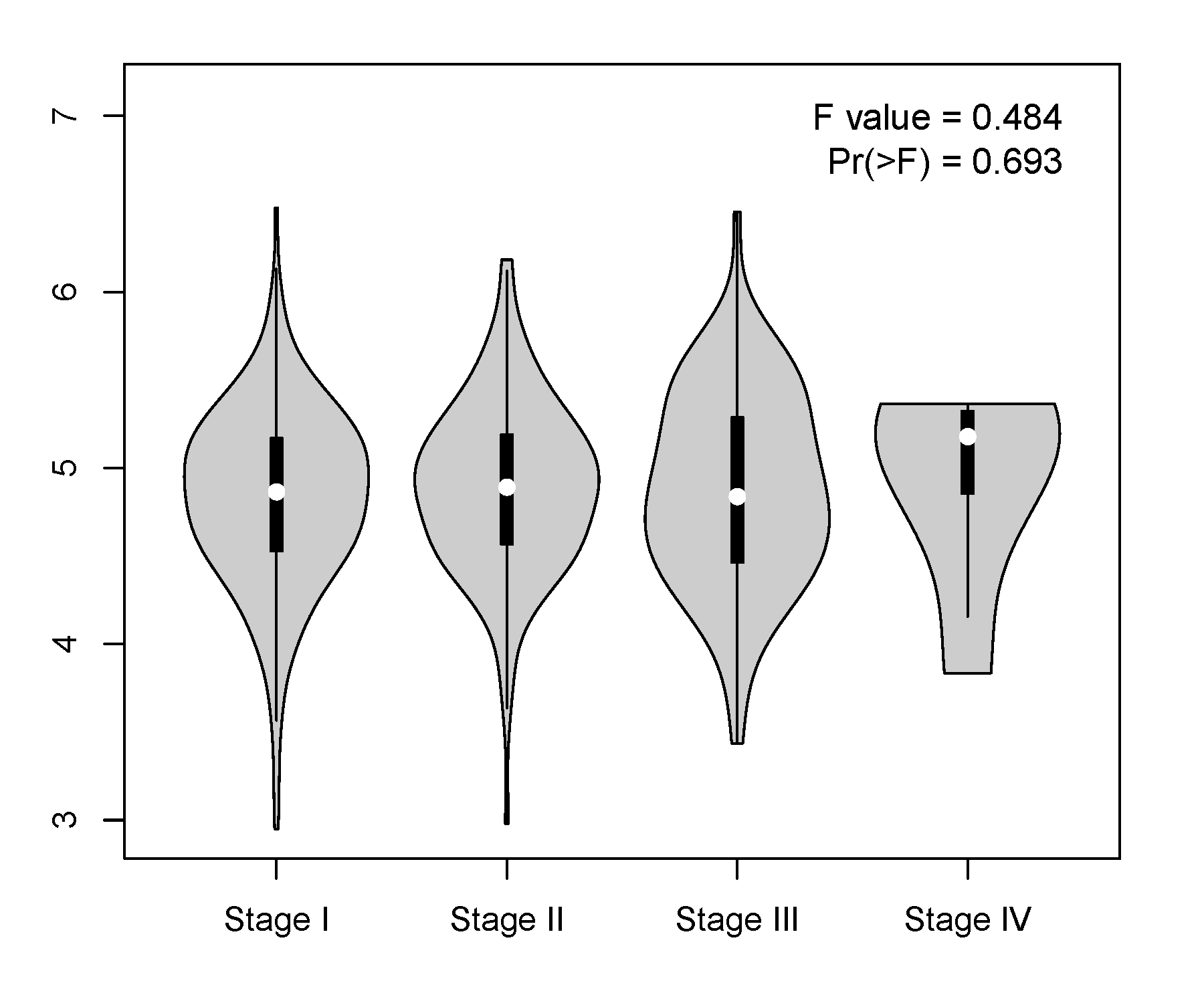

Supplement: Supplementary Figure 2 — PPI network for WTAP, YTHDC1, and YTHDF1. [file Image_2.TIF]

## Slide 1
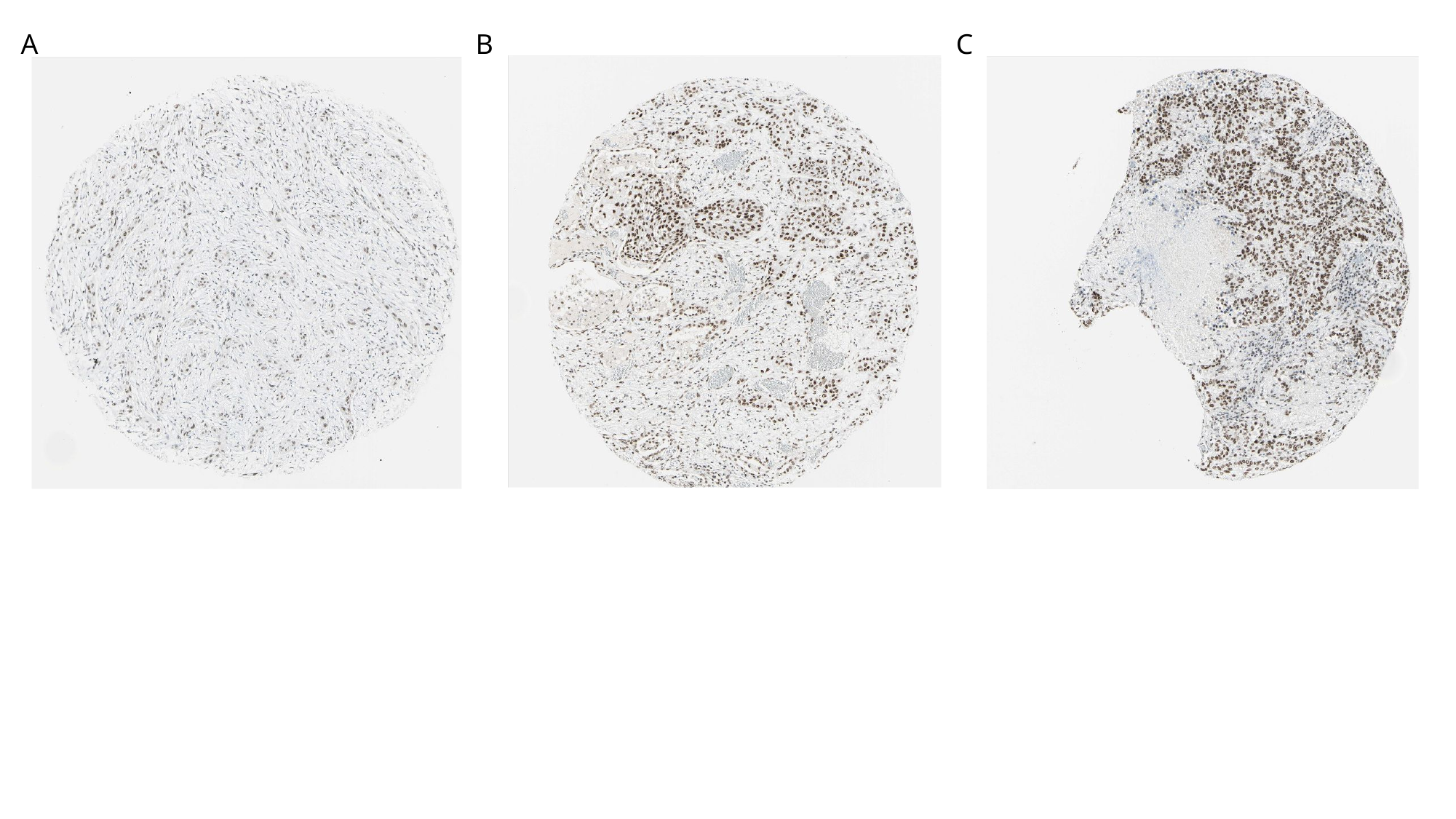

A
B
C

## Slide 2
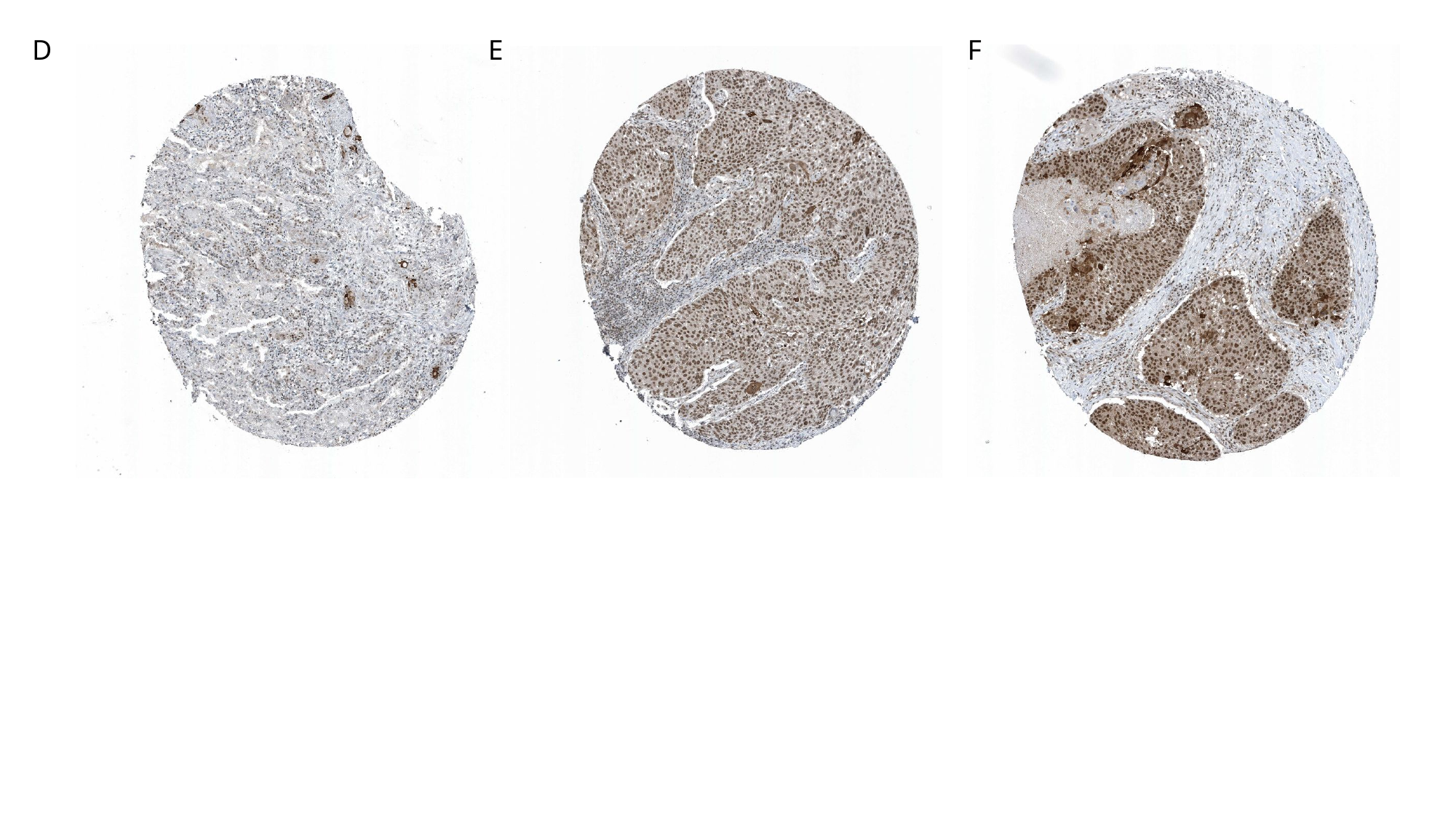

D
E
F

Supplement: Supplementary Figure 3 — The box plot for YTHDF1 in LUSC patients. [file Presentation_1.PPTX]

## Slide 1
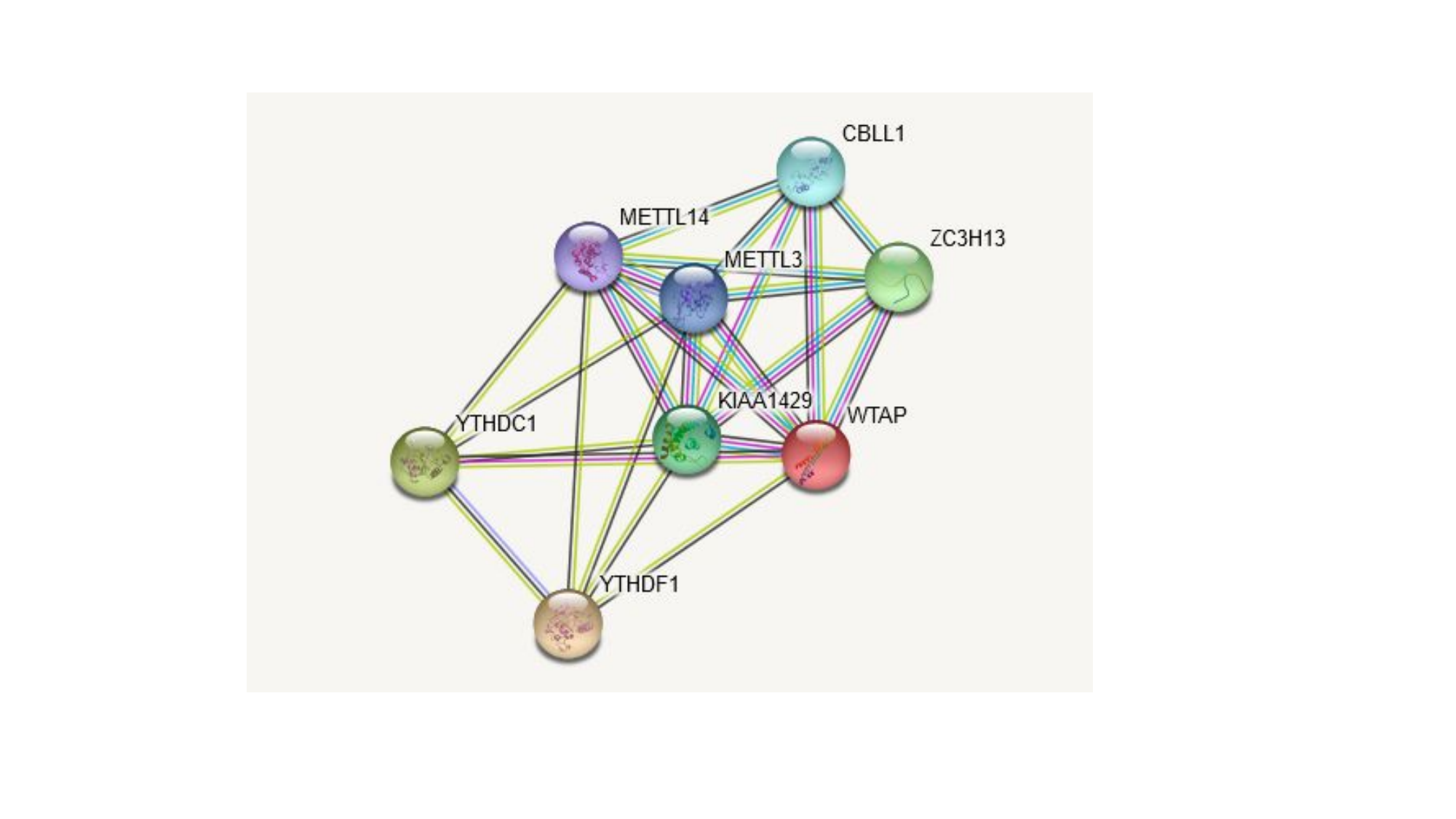

Supplement: Supplementary Figure 4 — The stage plot for YTHDF1 in LUSC patients. [file Presentation_2.PPTX]
