# Supplementary material for: Comprehensive Analysis of the Prognostic Role and Mutational Characteristics of m6A-Related Genes in Lung Squamous Cell Carcinoma
Source: Front Cell Dev Biol. 2021 Mar 25;9:661792. doi: 10.3389/fcell.2021.661792 (PMC8027321; doi:10.3389/fcell.2021.661792)
Supplement: Supplementary Table 1 — Baseline characteristics of the LUSC patients in TCGA database. [file Table_1.DOCX]

| Supplementary Table 1. Baseline characteristics | |
| --- | --- |
| Status |  |
| Alive | 285 |
| Dead | 216 |
| Age |  |
| Mean (SD) | 67.2 (8.6) |
| Median [MIN, MAX] | 68 [39,90] |
| Sex |  |
| FEMALE | 130 |
| MALE | 371 |
| Race |  |
| ASIAN | 9 |
| BLACK | 30 |
| WHITE | 349 |
| T |  |
| T1 | 50 |
| T1a | 24 |
| T1b | 40 |
| T2 | 172 |
| T2a | 87 |
| T2b | 34 |
| T3 | 71 |
| T4 | 23 |
| N |  |
| N0 | 319 |
| N1 | 131 |
| N2 | 40 |
| N3 | 5 |
| NX | 6 |
| M |  |
| M0 | 411 |
| M1 | 5 |
| M1a | 1 |
| M1b | 1 |
| MX | 79 |
| Stage |  |
| I | 3 |
| IA | 90 |
| IB | 151 |
| II | 3 |
| IIA | 65 |
| IIB | 94 |
| III | 3 |
| IIIA | 63 |
| IIIB | 18 |
| IV | 7 |
| Metastasis | 35 |
| Metastasis:Primary | 2 |
| Metastasis:Recurrence | 3 |
| Primary | 12 |
| Recurrence | 29 |
| Smoking |  |
| Non-smoker | 18 |
| Smoker | 471 |
| Radiation |  |
| No | 139 |
| Yes | 15 |
| Neoadjuvant |  |
| Yes | 6 |
| No | 494 |
